# DCSST Multi-Modular Equalization Scheme Based on Distributed Control

**DOI:** 10.3390/s21238125

**Published:** 2021-12-04

**Authors:** Fei Teng, Dezheng Kong, Zixuan Cui, Yuan Qin, Zhenghang Hao, Na Rong, Zhuo Chen

**Affiliations:** School of Electrical Engineering, Guizhou University, Guiyang 550025, China; tenfgeisnocu@163.com (F.T.); kdz_gzuedu@163.com (D.K.); czx1144051450@163.com (Z.C.); 17784197706@163.com (Y.Q.); haozhenghang@163.com (Z.H.); nrong@gzu.edu.cn (N.R.)

**Keywords:** DCSST, DAB, balance control, distributed control

## Abstract

As an important part of the DC micro-grid, DC solid-state transformers (DCSST) usually use a dual-loop control that combines the input equalization and output voltage loop. This strategy fails to ensure output equalization when the parameters of each dual active bridge (DAB) converter module are inconsistent, thus reducing the operational efficiency of the DCSST. To solve the above problems, a DCSST-balancing control strategy based on loop current suppression is presented. By fixing the phase-shifting angle within the bridge and adjusting the phase-shifting angle between bridges, the circulation current of each DAB converter module is reduced. Based on the double-loop control of the DAB, five controllers are nested outside each DAB submodule to achieve distributed control of the DCSST. The proposed control strategy can reduce the system circulation current with different circuit parameters of the submodules, ensure the balance of input voltage and output current of each submodule, and increase the robustness of the system. The simulation results verify the validity of the proposed method.

## 1. Introduction

**Figure 1:**
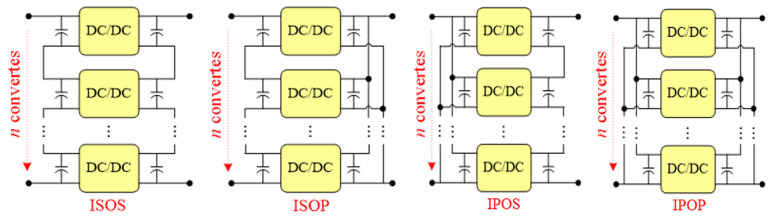
Scheme of Multiple Modular DCSST.

Dual active bridge DC-DC converters [1,2] are widely used in the generation of photovoltaic power [3], wind power grid connects [4], electric vehicles [5], and uninterruptible power supply equipment because of advantages such as electrical isolation, modular high-voltage and ultra-high-voltage resistance, high power density, bidirectional power transmission, and easy realization of soft switching. Traditional power-frequency transformers can meet the requirements of electrical isolation and voltage matching [6,7], but they are huge, heavy, and unfriendly to the environment, and discontinuous regulation and comprehensive control functions for the voltage and current disenable them gradually to meet the needs of the rapid development of society. Under this condition, the power electronic transformer has attracted the attention of experts and scholars at home and abroad because of advantages such as electrical isolation, voltage conversion, and compensation of reactive power [8]. To further improve the power level and power density, a power electronic transformer (PET) drive system is widely used, including a single-phase cascaded multilevel rectifier DAB converter. This paper focuses on a DAB converter with parallel output. As the middle stage of the three-stage power electronic transformer, the DAB connects the inverter circuit at the input end with the rectifier circuit at the output end through a high-frequency transformer to realize the purpose of power transmission and energy transmission. In the field of DC/DC converters, multi modularization is a typical scheme to improve the voltage and power level of the converter, such as through input-series—output-parallel (ISOP), input-series—output-series (ISOS), input-parallel—output-parallel (IPOP), and input-parallel—output-series (IPOS) schemes, as shown in Figure 1.

However, the discussion of multiple modular schemes for the DCSST mostly takes a unidirectional DC/DC converter as the basic unit, such as a buck—boost, forward—backward, push—pull, half-bridge, one-sided rectifier full-bridge structure, etc. These applications can increase the voltage and power level, but the technical requirements are not met in the case of bidirectional power flow. Various input-series—output-shunt combinations of DC/DC converters have been studied in the past [9,10]. However, these ISOP topologies are based on unidirectional DC/DC converter units. These types of DC/DC converters can be used for DC load, energy storage, and distributed power supply access in low-voltage DC microgrids. However, it cannot be used as a link of energy transformation between the high-voltage DC (HVDC) distribution network and the HVDC microgrid. It is difficult to meet the requirements of bidirectional power transmission in a DC power grid. Document [11] puts forward the concept of a DCSST based on the series-parallel connection of a DAB converter. By using the DAB unit, the flexibility of the variable voltage range and two-way power adjustment of the DCSST is greatly improved. Document [12] applies a DAB-based DCSST to flexible medium-voltage DC distribution systems with multiple distributed power sources. For the control method of the DCSST, the decoupling control strategies of the output voltage loop and input-equalizing voltage loop of the DCSST are obtained by different deduction methods in the literature [13,14]. However, all the above-mentioned papers propose control strategies under the condition that the transmission efficiency of each module in the DCSST is the same. In practice, due to the different power parameters of each module, the transmission efficiency of each submodule may be uneven, so that the DCSST with the above-proposed control strategy cannot guarantee both input voltage and output current equalization. When the DCSST output is not equalized, the power distribution among the modules in the DCSST will be unbalanced, and the output current of a single DAB will be too large or even exceed the rated current, which will increase the probability of damage to a single DAB and make the DCSST not operate normally. At the same time, the loop current generated will increase the system loss and reduce the operational efficiency of the whole system. For the uneven transmission power of each DCSST submodule, the literature [15] calculates the actual power and compares it with the power reference value by sampling the obtained voltage and current to achieve the purpose of controlling the power equalization. However, additional current sensors are required, which increases the design cost. In [16], a power-balancing control method without a current sensor is proposed to reduce the design cost. Studies [17,18] proposed a virtual power balancing control method and a direct power balancing control method, respectively. However, the studies [15,16,17,18] all control the submodule transmission power equalization by adjusting the transmission power to achieve the output current equalization, which is more complicated to calculate. Reference [19] used virtual impedance control to change the output characteristics of the parallel system without consuming additional power to achieve regulation of the output current. References [20,21] propose to add virtual impedance to regulate the output uniform current based on the closed-loop control of the inverter, but it increases the output impedance of the whole system and affects the output characteristics of the whole system.

By using a bidirectional DC/DC converter unit, the multi-modular scheme can also be applied to a DC transformer with bidirectional power flow capability. To provide electrical isolation and improve power density, the DC/DC converter unit needs to adopt topology with high-frequency isolation. Compared with the device series and modular multilevel schemes, the multi-modular scheme is more flexible. It can meet various application requirements through series or parallel schemes. It can adjust the system capacity and provide redundancy by increasing or decreasing modules. In addition, the high-frequency isolation transformer is dispersed, the capacity is small, and the manufacturing difficulty is small.

Regarding the above background, the main research objective for DCSSTs in this paper is ISOP-DAB multiple modular technology schemes. Firstly, the structure and operation mode of the DAB is described, then the operation mode and transmitted power characteristics of the DCSST are analyzed, and the distributed control strategy of the DCSST is proposed in combination with the dual-loop control of the DAB. The conventional frequency converter is abandoned as the coupler between the two bridges of the converter and a high-frequency transformer are used, which further improves the power density, modalities, cost reduction, and noise reduction of the system. The method used improves the robustness of the modular DCSST. The distributed control scheme proposed in this paper can effectively reduce the system loop current, improve the power balance, and adapt to higher voltage levels without affecting the original output characteristics of the DCSST, which is suitable for high-power, high-voltage DC transmission applications. Finally, the effectiveness of the proposed method is verified by simulation.

## 2. DAB Structure and Operating Mode

**Figure 2:**
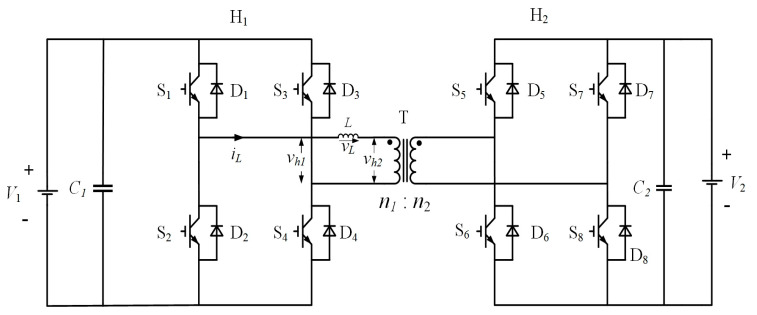
DAB Extension Diagram.

As shown in Figure 2, the circuit topology of the DAB converter consists of two full-bridge circuits and a high-frequency transformer, which is completely symmetrical in structure. It has advantages of electrical isolation, bidirectional energy flow, modular structure, and easy parallel connection [22].

**Figure 3:**
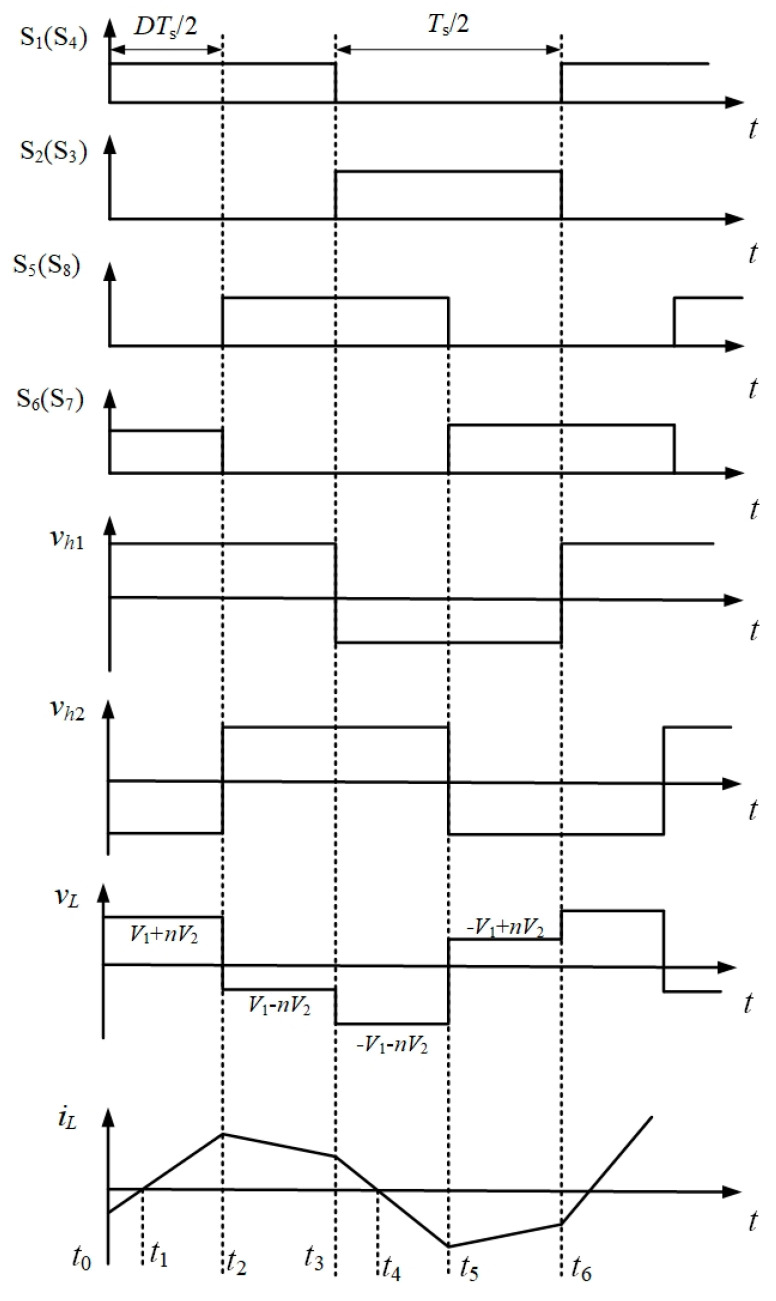
Working state of DAB converter under single-phase-shift control.

DAB converters have many control methods, the most basic of which is using a single-phase-shift control. SPS control means that both the primary side full bridge $H_{1}$ and the secondary side full bridge $H_{2}$ use a pulse-width-modulation (PWM) wave with a 50% duty cycle as the control signal of the switch tube. However, there is an angle between the control signals of the two full bridges, which is called the phase-shift angle. By changing the phase-shift angle, voltage, and current on the primary side auxiliary inductor, VL can be changed to the direction of energy flow and the magnitude of the secondary side voltage. The working state of the DAB circuit with a single-phase-shift angle is shown in Figure 3 [23,24,25].

## 3. DC Transformer Based on ISOP-DAB

### 3.1. Working Mode

**Figure 4:**
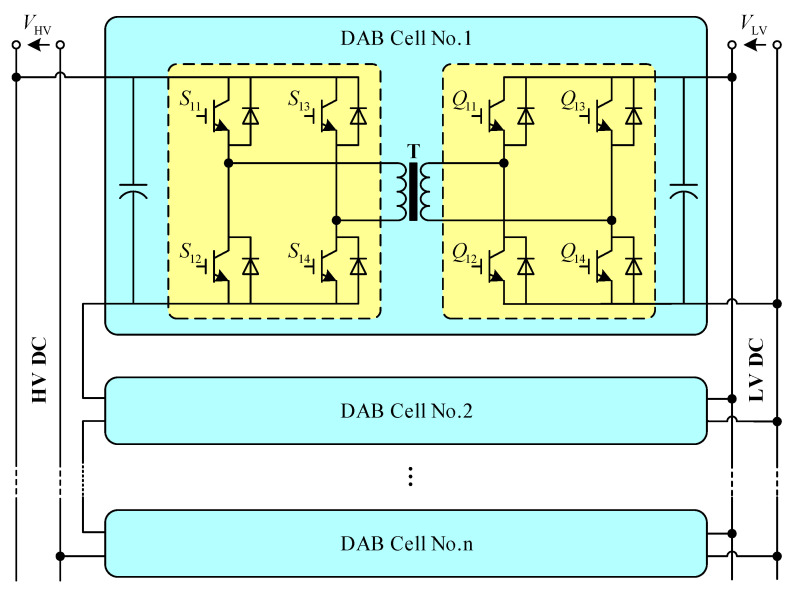
DCSST Topology Based on ISOP-DAB.

Figure 4 is the topology of the DCSST based on the ISOP-DAB proposed in this paper.

In traditional AC magnetic transformers, the voltage transformation is mainly determined by the winding turn ratio, while the power flow is determined by the load. In the DCSST, three modes are designed: HV voltage control mode, LV voltage control mode, and power control mode.

Mode 1: HV voltage control mode

In HV voltage control mode, the LVDC bus voltage is fixed, the HVDC bus voltage is controlled by the DCSST, and the power flow size and direction are determined by the load.

Mode 2: LV voltage control mode

In LV voltage control mode, the HVDC bus voltage is fixed, and the LVDC bus voltage is controlled by the DCSST. In addition, the DCSST needs to control the voltage balance at the series end and the power balance at the parallel end of each DAB unit. The size and direction of the power flow are determined by the load.

Mode 3: power control mode

In power control mode, the high- and low-voltage bus voltage are fixed, and the DCSST controls the size and direction of the power flow and the voltage balance at the series end and the power balance at the parallel end of each DAB unit.

Based on the above analysis, the DCSST not only achieves voltage-level transformation and electrical isolation of high and low voltage, but also achieves active control of the voltage, current, and power. The DCSST has fewer transforming steps than the ACSST, so it is more efficient. With the rapid development of DC transmission and distribution, DCSST has great application prospects in DC distribution.

### 3.2. Transmission Power Characteristics

**Figure 5:**
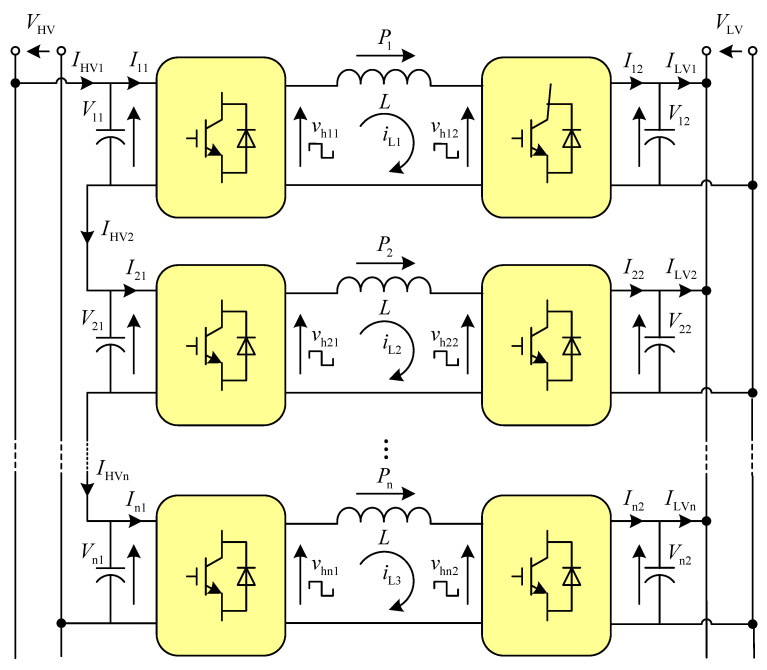
The Average Model Equivalent Circuit of DCSST.

The average model equivalent circuit of a DCSST is given in the following Figure 5.

As shown in Figure 5, V_HV_ and V_LV_ are the DCSST’s high-voltage and low-voltage bus voltage; $I_{HVi}$ and $I_{LVi}$ are the average currents at the HVDC and LVDC ends of each DAB unit; $V_{i1}$ and $V_{i2}$ are the average voltages of each DAB unit at the HVDC and LVDC ends; $I_{i1}$ and $I_{i2}$ are the average currents of the entire bridge at the HVDC and LVDC ends for each DAB unit; $V_{hi1}$, $V_{hi1}$ is the AC terminal voltage of the full-bridge converters for each DAB unit; $I_{Li}$ is the AC terminal current of the full-bridge converter; Pi is the average transmission power of each DAB unit; in the above variables, i = 1, 2, ... n.

When using dual-phase-shifting (DPS) control for each DAB unit in the DCSST, in addition to the external phase-shifting angle $D_{i2}$ between full-bridge converters on both sides of the DAB, there is an internal phase-shifting angle $D_{i1}$ within each full-bridge converter. The transmission power of the DAB is controlled by controlling the phase shift between $V_{hi1}$ and $V_{hi2}$, and then the transmission power of the DCSST is controlled.

For each DAB unit, the transmission power can be expressed as:

(1) $$P_{i}=\frac{n_{T}V_{i1}V_{i2}}{8f_{s}L}\left\{ \begin{array}{ccc} \left[4D_{i2}(1-D_{i2})-2D_{i1}^{2}\right] & & D_{i1}\leq D_{i2}\\ \left[4D_{i2}(1-D_{i2})-\frac{1}{2}D_{i1}^{2}\right] & & D_{i1}>D_{i2} \end{array}\right.$$

The value of 0 < $D_{i1}$, $D_{i2}$< 1, $f_{s}$ is the switching frequency, $n_{T}$ is the transformer ratio, and L is the sum of the series auxiliary inductance and leakage inductance for high-frequency transformer *T*.

For the DCSST, each DAB unit has the same current on the series side and the same voltage on the parallel side. We can see:

(2) $$\left\{ \begin{array}{l} V_{HV}=V_{11}+V_{21}+\cdot \cdot \cdot +V_{n1}\\ I_{HV}=I_{HV1}=I_{HV2}=\cdot \cdot \cdot =I_{HVn}\\ V_{LV}=V_{12}=V_{22}=\cdot \cdot \cdot =V_{n2}\\ P_{\mathrm{DCSST}}=P_{1}+P_{2}+\cdot \cdot \cdot +P_{n} \end{array}\right.$$

$P_{\mathrm{DCSST}}$ is the transmission power of the DCSST. The control methods and parameters of each DAB are consistent, and there are

(3) $$\left\{ \begin{array}{l} D_{1}=D_{11}=D_{21}=\cdot \cdot \cdot =D_{n1}\\ D_{2}=D_{12}=D_{22}=\cdot \cdot \cdot =D_{n2} \end{array}\right.$$

From the above analysis, we can see:

(4) $$P_{\mathrm{DCSST}}=\frac{n_{T}V_{HV}V_{LV}}{8f_{s}L}\left\{ \begin{array}{lll} \left[4D_{2}(1-D_{2})-2D_{1}^{2}\right] & & D_{1}\leq D_{2}\\ \left[4D_{2}(1-D_{2})-\frac{1}{2}D_{1}^{2}\right] & & D_{1}>D_{2} \end{array}\right.$$

The maximum transmission power of the DCSST can be obtained at $D_{1}$ = 0, $D_{2}$= 0.5, and the maximum value is

(5) $$P_{\mathrm{DCSST}\_\max}=\frac{n_{T}V_{HV}V_{LV}}{8f_{s}L}$$

**Figure 6:**
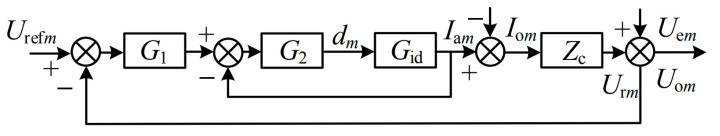
Dual-Loop Control Block Diagram of DAB.

When the DAB uses a double closed-loop control strategy [26], the control block diagram is shown in Figure 6.

Figure 6: $U_{refm}$ is the reference voltage; $U_{rm}$ is the ideal output voltage for DAB; $U_{em}$ is the deviation voltage caused by a drop in the dead-zone voltage in the DAB switch. $G_{1}$ is the voltage outer-loop regulator and $G_{2}$ is the current inner-loop regulator.

**Figure 7:**
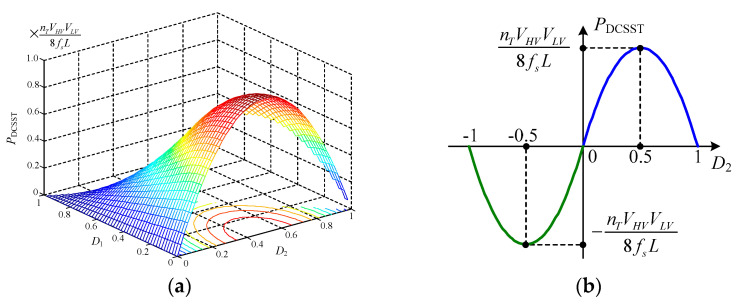
Transmission power model for DCSST. (**a**) Global Model. (**b**) Internal Shift Ratio 0.

The transmission power model of the DCSST is shown in the following Figure 7.

From the power model, it can be seen that when the control and parameters of each DAB unit are the same, the transmission power model of the DCSST and DAB is also basically the same. When the internal shift is fixed, the maximum transmission power is obtained at $D_{2}$ = 0.5. When $D_{2}$ is less than or equal to 0.5, the transmission power $P_{\mathrm{DCSST}}$ increases with the outward shift ratio. Furthermore, the power models’ symmetry is about $D_{2}$ = 0.5. The main difference between the DCSST and DAB power models is that the high-voltage DC voltage is the sum of the series voltage of all DAB units.

## 4. Voltage and Power Balance Control

With the DCSST, the most important thing is to control the input voltage balance of each DAB series unit at the HVDC end and the output current balance of each DAB parallel unit at the LVDC end. It should be noted that, unlike AC distribution networks, in DC distribution networks, the energy exchange between low-voltage and high-voltage distribution buses should be bidirectional due to the presence of distributed power, so the energy flow control of the DCSST is bidirectional. The two-way interactive function is the main advantage and trend of the development of modern, flexible DC distribution networks.

Ignoring the system’s power loss, the average current flowing through the capacitance in a switching cycle is 0 under a steady state. There is,

(6) $$\left\{ \begin{array}{l} I_{HVi}=I_{i1}\\ I_{LVi}=I_{i2} \end{array}\right.$$

The transfer power of the DAB unit is equal at input and output, so

(7) $$P_{i}=V_{i1}I_{i1}=V_{i2}I_{i2}$$

From the above analysis, we can see that

(8) $$V_{11}=V_{21}=\cdot \cdot \cdot =V_{n1}\Leftrightarrow P_{1}=P_{2}=\cdot \cdot \cdot =P_{n}$$

Equation (8) shows that the voltage balance at the series end of each DAB unit is equivalent to the power balance at the parallel end. Therefore, in DCSST, only one of the voltage or power balances needs to be controlled.

**Figure 8:**
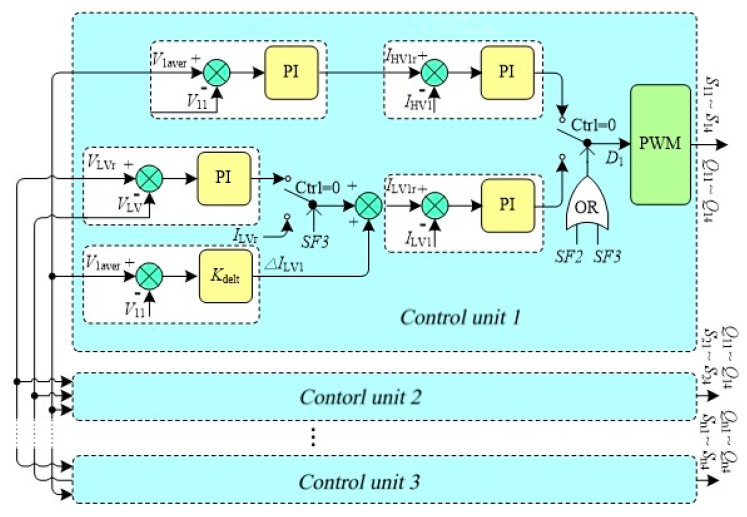
Distributed control and management strategy for DCSST.

To improve the dynamic performance and stability of the system, a distributed control and management strategy for the DCSST is presented in Figure 8.

Each subunit has the same control model, which is composed of an HV voltage controller, HV current controller, LV voltage controller, LV current controller, and balance controller. In the diagram, SF1~SF3 is a binary logic control signal, which represents the HV voltage control mode, LV voltage control mode, and power control mode in turn. When it is 1, it represents the corresponding enabling mode, otherwise, it represents the prohibiting mode. In the figure, SF1~SF3 is a binary logic control signal, which represents the HV voltage control mode, LV voltage control mode, and power control mode in turn. When it is 1, it represents the corresponding enabling mode, otherwise, it represents the prohibiting mode.

In the HV voltage control mode, the low-voltage bus voltage is fixed, the HV voltage and current controller are selected, and each DAB controls the respective high-voltage terminal voltage and ensures that the respective voltage is equal to distribute the load equally. The power flow size and direction are determined by the load. In the LV voltage control mode, the high-voltage bus voltage is fixed, the LV voltage, current controllers, and balance controllers are turned on, and the low-voltage bus voltage is controlled by the DCSST. In addition, the DCSST controls the voltage balance of each DAB unit in series. The size and direction of the power flow are determined by the load. In the LV voltage control mode in Figure 7, the error between the bus voltage $V_{LV}$ and reference value $V_{LVr}$ is fed into the PI controller, and the output of the PI controller is used as the uniform reference value for the internal current loop controllers of each DAB unit.

The balance controller collects the DC voltage $V_{i1}$ of each DAB series unit, calculates the average voltage $V_{1aver}$ according to Equation (9), and calculates the corrected amount $\Delta_{ILVi}$ of the reference current of each DAB unit based on the difference between $V_{1aver}$ and $V_{i1}$. The sum of the output and corrected amount $\Delta_{ILVi}$ of the LV voltage controller is the current reference value of each DAB unit.

(9) $$V_{1aver}=(V_{11}+V_{21}+\cdot \cdot \cdot +V_{n1})/n$$

There is no integral parameter in the calculation of the correction $\Delta_{Ii2}$, only the proportional control parameter $K_{delt}$.

(10) $$\left\{ \begin{array}{l} \Delta I_{i2}=K_{delt}(V_{1aver}-V_{i1})\\ \Delta I_{12}+\Delta I_{22}+\cdot \cdot \cdot +\Delta I_{n2}=0 \end{array}\right.$$

According to Equation (10), the sum of current corrections is zero, so the control of the HV bus voltage equalization does not affect the control of the HV bus voltage.

In power control mode, the DCSST control state is similar to the LV voltage control mode, except that the high- and low-voltage bus voltage are fixed in power control mode, only the LV current controller and balance controller are selected, and the uniform reference value of the LV current controller is calculated directly according to the specified power transmission size.

In addition, to simplify control, in the design of the control strategy, the outward shift compared to Di2 is calculated by the control system feedback to control the size and direction of the transmission power. Di1 is set to a constant value to reduce the circular power of the system to a certain extent.

## 5. Simulation and Analysis

**Figure 9:**
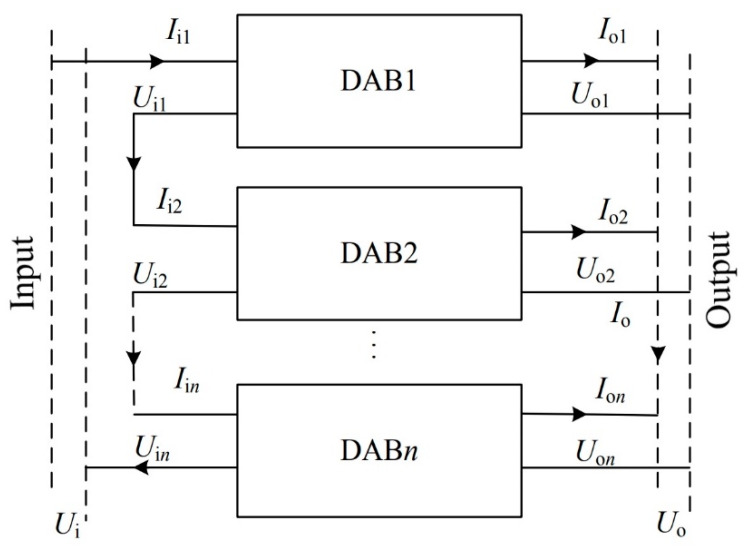
Structure of DCSST.

To verify the validity of the control strategy proposed in this paper, the model of the DCSST [27] shown in Figure 9 was built in MATLAB, and the control strategy based on virtual impedance was applied.

In Figure 9, $U_{i}$ and $U_{o}$ represent the input and output bus voltage of the DCSST. $U_{ik}$ and $U_{ok}$ represent the input and output voltage of each DAB module. $I_{ik}$ and $I_{ok}$ represent the current of each DAB module on the input and output sides. $I_{i}$ and $I_{o}$ represent the current on the input and output sides of the DCSST, where k = 1, 2, ..., n

The simulation parameters are shown in Table 1.

**Figure 10:**
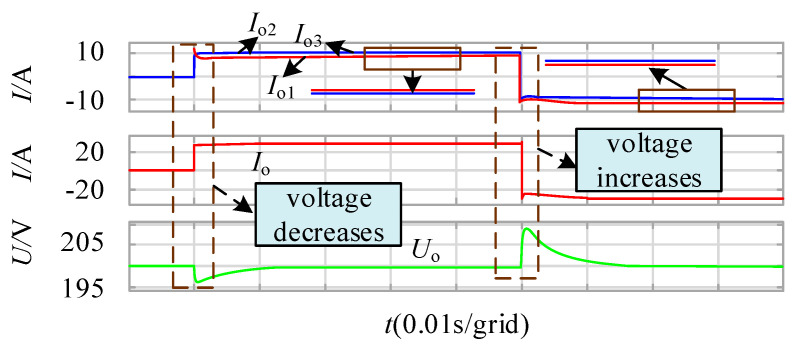
Output current and output voltage with different parameter diagram of DCSST.

The output current imbalance caused by different parameters of the DAB module 1 under the traditional modular control strategy of the DC transformer is verified. The simulation result’s waveform is shown in Figure 10.

It can be seen from the diagram that at 0.01 s, the output side load of the DCSST increases, and the DC bus voltage decreases. Due to the different parameters of modules 2 and 3 compared to module 1, the output current of module 1 is different from modules 2 and 3, and the output current is not balanced. At 0.06 s, the output side load of the DCSST decreases, the DC bus voltage increases, and the output current of module 1 is different from that of modules 2 and 3. The output current is not balanced.

**Figure 11:**
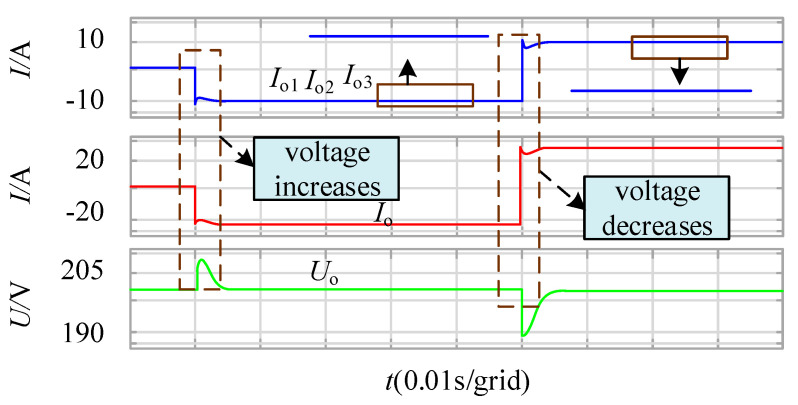
Output current and voltage waveform after power balance control.

As shown in Figure 11, the load decreases and increases at 0.01 s and 0.06 s, respectively. The bus voltage on the output side rises and decreases through fluctuations. It can be seen from the figure that the main circuit parameters of module 1 are different from other modules, but the output current is the same after adding power balance control. At the same time, the output voltage remains stable at 200 V.

**Figure 12:**
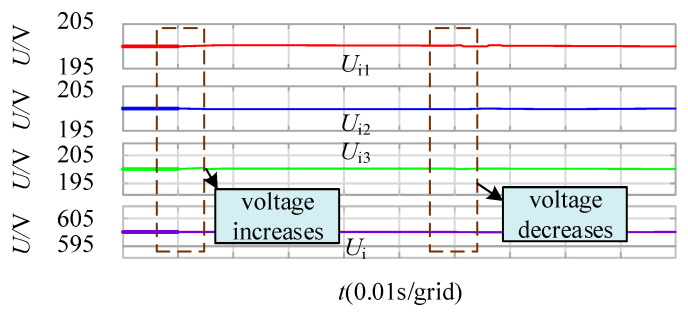
Input voltage waveform after power balance control.

Figure 12 shows the input voltage waveforms of each module with different main circuit parameters. The total input voltage of each module is stable at 600 V, and the input voltage of each module is stable at 200 V. It can be seen that the control strategy proposed in this paper can not only ensure output current sharing and output voltage stability, but also ensure input voltage sharing.

## 6. Conclusions

For the proposed distributed control and management strategy of the DCSST based on ISOP–DAB, this paper first introduces the structure and working mode of the DAB, the working mode and transmission power characteristics of the DCSST, and the unbalanced output current caused by different submodule parameters. A multi-module DCSST scheme based on ISOP–DAB is presented, and the principle of its distributed control and management strategy is demonstrated. The unbalanced output current caused by different power parameters of each DAB module is solved. The DCSST can balance the output current with different main circuit parameters of each module, and at the same time, when the DC bus voltage on the output side changes abruptly, the output voltage can be stabilized. The simulation results show that the proposed control strategy can achieve output current balance based on output voltage stability and input voltage balance.

At the same time, there are still some unresolved problems in this paper, such as minimizing the return power, soft switch, and long simulation time, which will be the next research topic of the author.

## Figures and Tables

**Table 1:** DCSST simulation parameters.

Parameter Name/Unit	Input Voltage U/V	Output Voltage U/V	Inducance L/μH	Internal Resistance R/Ω	Capatance C/μ F	Transformation Ratio n	Switching Frequency fs/kHz
DAB1	200	200	17	0.3	1000	1	50
DAB2	200	200	10	0	1000	1	50
DAB3	200	200	10	0	1000	1	50
